# Optimizing Drug-Coated Balloon Outcomes: The Evolving Role of Vessel Preparation in Coronary and Peripheral Artery Interventions

**DOI:** 10.3390/life16101756

**Published:** 2026-10-20

**Authors:** Sara Malakouti, Sandeep Basavarajaiah, Nihit Shah, Bernardo Cortese

**Affiliations:** 1 DCB Academy, Cleveland, OH 44106, USA; dr.saramalakouti@gmail.com (S.M.);; 2 Cardiovascular Research Group, Fondazione Ricerca e Innovazione Cardiovascolare, 20143 Milano, Italy; 3 Department of Cardiology, Heartlands Hospital, University Hospitals Birmingham NHS Foundation Trust, Birmingham B9 5SS, UK; 4 Case Western Reserve University, Cleveland, OH 44106, USA

**Keywords:** drug-coated balloon, vessel preparation, lesion modification, coronary intervention, peripheral artery disease, intravascular imaging, calcium modification, intravascular lithotripsy, atherectomy, leave-nothing-behind strategy

## Abstract

**Highlights:**

**Why is vessel preparation important in DCB therapy?**

- Vessel preparation is a treatment determinant, not merely a procedural step, in DCB therapy.

**What are the effects of advanced vessel preparation?**

- Advanced preparation improves procedural success more consistently than long-term clinical outcomes.

**How can vessel preparation be optimized?**

- Lesion-specific, imaging-guided preparation may enable a shift from device selection to treatment-substrate optimization.

**Abstract:**

Drug-coated balloon (DCB) therapy differs fundamentally from stent-based revascularization since the vessel itself becomes the treatment substrate. Consequently, vascular preparation is an important component of DCB therapy. This review therefore examines contemporary vessel preparation before DCB therapy across coronary and peripheral interventions, integrating lesion morphology, procedural outcomes, intravascular imaging, and clinical follow-up. Across vascular beds, the objective is not maximal plaque disruption, but sufficient modification of the dominant mechanical limitation to achieve predictable expansion, controlled vessel injury, and effective balloon–vessel wall contact. Subsequent failure after coronary DCB therapy has been associated with residual plaque burden and calcium morphology. Specialty balloons, atherectomy and intravascular lithotripsy have been associated with improved procedural success, lumen expansion, and calcium modification, with lower rates of flow-limiting dissection or bailout stenting in selected settings. Randomized evidence has generally demonstrated more consistent procedural than long-term clinical benefits. Moreover, much of the evidence supporting advanced calcium-modification strategies derives from stent-based interventions and cannot be directly extrapolated to DCB-only therapy. These findings favor a lesion-specific, preparation-dependent approach rather than routine escalation to increasingly aggressive technologies. Conventional balloons may be sufficient for mechanically responsive lesions, whereas scoring or cutting balloons, atherectomy, or intravascular lithotripsy should be selected according to lesion morphology and the specific mechanical limitation that persists after initial preparation. Intravascular imaging may facilitate objective assessment of a “DCB-ready” vessel, although validated preparation thresholds remain undefined. The question is thus not which preparation device is preferable but whether the vessel has been adequately prepared to receive the drug. Future studies should go beyond device-to-device comparisons and assess whether predefined, imaging-based preparation targets improve drug delivery, vessel healing, and durable clinical outcomes. The next step toward precision DCB therapy across coronary and peripheral disease may be to shift the focus from device selection to treatment-substrate optimization.

## 1. Introduction

Drug-coated balloons (DCB) have become an important part of “leave-nothing-behind” revascularization in both coronary and peripheral artery disease. Unlike stent-based therapy, DCB treatment relies on adequate lesion preparation as there is no scaffolding to address recoil and dissection creating by balloon dilatation. Sufficient plaque modification, acute luminal gain, and good balloon–vessel wall contact may facilitate drug transfer and contribute to an adequate treatment substrate, although direct evidence linking the extent of lesion preparation to tissue drug delivery remains limited. The key question, therefore, is not simply whether a lesion can be treated with a DCB, but whether better vessel preparation is associated with better procedural and longer-term outcomes [1,2,3].

Heavily calcified and morphologically complex coronary lesions can impede adequate balloon expansion and increase procedural risk, often necessitating adjunctive lesion-preparation tools such as specialty (cutting/scoring or ultra–high-pressure) balloons, atherectomy, and intravascular lithotripsy (IVL). These modalities achieve plaque modification through complementary mechanisms; balloon based plaque scoring/disruption and controlled neointimal modification, atheroablative debulking, and IVL-mediated calcium fracture and are now increasingly integrated into contemporary lesion-preparation algorithms across both coronary and peripheral endovascular practice [4,5]. Effective vessel preparation may be particularly important when DCB therapy is planned, because DCBs provide no permanent mechanical scaffolding. In this context, the value of lesion preparation extends beyond achieving acute luminal gain; it may also facilitate successful definitive DCB treatment, minimize the need for crossover to stenting or other adjunctive therapy, and ultimately promote durable vessel patency. However, the available evidence remains heterogeneous across coronary and peripheral vascular territories, and much of the current understanding of lesion preparation has been derived from stent-based interventions rather than from DCB-specific studies [6].

### 1.1. Review Methodology

This manuscript was designed as a structured contemporary narrative review. MEDLINE/PubMed and Embase were searched from database from 2013 through 2026 using combinations of terms related to “drug-coated balloon,” “DCB,” “vessel preparation,” “lesion preparation,” “lesion modification,” “coronary intervention,” “peripheral artery disease,” “intravascular imaging,” “IVUS,” “OCT,” “calcium modification,” “intravascular lithotripsy,” “IVL,” “atherectomy,” “scoring balloon,” “cutting balloon,” and “restenosis.” Eligible publications included randomized controlled trials, prospective registries, retrospective cohort studies, observational studies, and systematic reviews or network meta-analyses addressing vessel or lesion preparation before DCB therapy, preparation quality, lesion morphology, imaging-based assessment, procedural outcomes, or clinical follow-up in coronary or peripheral interventions. Studies that did not address these domains were excluded. Studies focused exclusively on stent-based interventions were included only when they provided relevant mechanistic or indirect evidence regarding vessel preparation, calcium modification, or vessel mechanics; such evidence was interpreted separately from DCB-specific evidence. The literature was synthesized according to vascular territory, lesion morphology, preparation strategy, imaging assessment, and DCB-specific procedural and clinical outcomes, with interpretation weighted according to study design and level of evidence.

### 1.2. Scope and Objectives

An important question, therefore, is which lesion-preparation strategy is best suited to a given lesion and whether more effective plaque modification translates into improved long-term clinical outcomes. In this review, we examine the contemporary evidence supporting specialty balloons, atherectomy, and IVL in coronary and peripheral interventions. By integrating clinical outcomes with lesion morphology and the mechanistic determinants of drug delivery, we aim to define when vessel preparation adds meaningful value to DCB therapy and whether optimizing the vessel before drug delivery can improve procedural success and long-term outcomes.

## 2. Why Vessel Preparation Influences Drug-Coated Balloon Success

Successful DCB therapy depends on two requirements: adequate acute vessel expansion and effective transfer of the antiproliferative drug to the vessel wall. Lesion morphology can influence both processes. Fibrotic and calcified tissue resist balloon expansion, moreover calcification reduces vessel compliance and may limit uniform balloon–wall contact, potentially compromising both luminal gain and drug delivery [7]. Vessel preparation should therefore aim to create a sufficiently expanded and stable vessel substrate while avoiding excessive vascular injury that would require crossover to DES.

The relationship between vessel preparation and DCB drug delivery is biologically plausible but remains incompletely established. Adequate lesion modification may improve drug transfer by promoting more uniform balloon–vessel wall contact; however, direct evidence remains limited. In a 2025 human cadaveric PAD model, plaque morphology strongly influenced paclitaxel deposition, with smoother lesions showing more uniform drug coverage than nodular lesions, while cutting-balloon preparation increased drug deposition when grooves were successfully created [8]. In clinical practice, the importance of the final treatment substrate is supported by the observational study of Konishi et al., in which greater residual plaque burden before coronary DCB treatment was associated with higher target-lesion failure, although tissue drug uptake was not measured [9]. Thus, vessel preparation may facilitate more consistent DCB drug transfer, but whether a specific preparation strategy or greater preparation intensity increases tissue drug uptake and improves long-term DCB outcomes remains uncertain.

Optical coherence tomography (OCT) data from Lee et al., provided a complementary lesion-level perspective. Among 328 de novo coronary lesions treated with DCB alone, target lesion failure (TLF) occurred in 31 lesions (9.5%) during a median follow-up of 460 days. Lesions associated with TLF had a substantially greater maximum calcium arc (215° vs. 104°) and were less frequently associated with medial dissection after treatment (16.1% vs. 60.9%). Each 90° increase in maximum calcium arc was associated with an increased risk of TLF (HR, 1.34; 95% CI, 1.05–1.72; *p* = 0.02), while the absence of medial dissection was strongly associated with TLF (HR, 8.24; 95% CI, 3.15–21.6; *p* < 0.001). Patients with TLF were also more likely to require hemodialysis (29.0% vs. 10.8%). These observations reinforce the importance of calcium burden and post-treatment vessel-wall morphology as potential determinants of DCB durability. Importantly, however, the association between medial dissection and lower TLF should not be interpreted as evidence that deeper dissections should be intentionally created [10].

Recently, in a pooled analysis of 2128 ISR lesions treated with DCB angioplasty, Khialani et al. compared specialty balloon (cutting or scoring) with regular balloon lesion preparation. Specialty balloon use was related with less residual stenosis following DCB angioplasty (3.3% vs. 6.4%; *p* = 0.015) and a reduced rate of MACE over a mean follow-up of 734 ± 457 days (8.0% vs. 18.0%; *p* = 0.006). This correlation remained significant after multivariable adjustment (HR, 0.45; 95% CI, 0.24–0.84; *p* = 0.013), suggesting that specialty balloon–based lesion preparation may improve procedural and longer-term results in patients with ISR following DCB treatment [11].

The importance of vessel preparation is also reflected in the mechanisms underlying procedural failure during DCB-only PCI. In a single-center cohort examining the need for bailout treatment, flow-limiting dissection accounted for 70.7% of procedural failures, followed by residual stenosis >30% (27.6%) and vascular closure (1.7%). Prior coronary artery bypass grafting (CABG) (OR, 4.29; 95% CI, 1.70–10.80; *p* = 0.002), proximal lesion location (OR, 3.00; 95% CI, 1.46–6.14; *p* = 0.003), and diffuse disease (OR, 2.18; 95% CI, 1.15–4.14; *p* = 0.018) independently predicted the need for bailout treatment, whereas prior PCI (OR, 0.44; 95% CI, 0.23–0.81; *p* = 0.009) and lipid-lowering therapy (OR, 0.42; 95% CI, 0.20–0.92; *p* = 0.029) were associated with lower risk. The resulting Bailout Risk Score demonstrated moderate discrimination (AUC, 0.734; optimism-corrected AUC, 0.704; 95% CI, 0.675–0.735) with good calibration. Collectively, these findings suggest that lesion complexity and the ability to achieve an acceptable acute angiographic result are central determinants of whether a stentless DCB strategy can be completed successfully [12].

**Figure 1:**
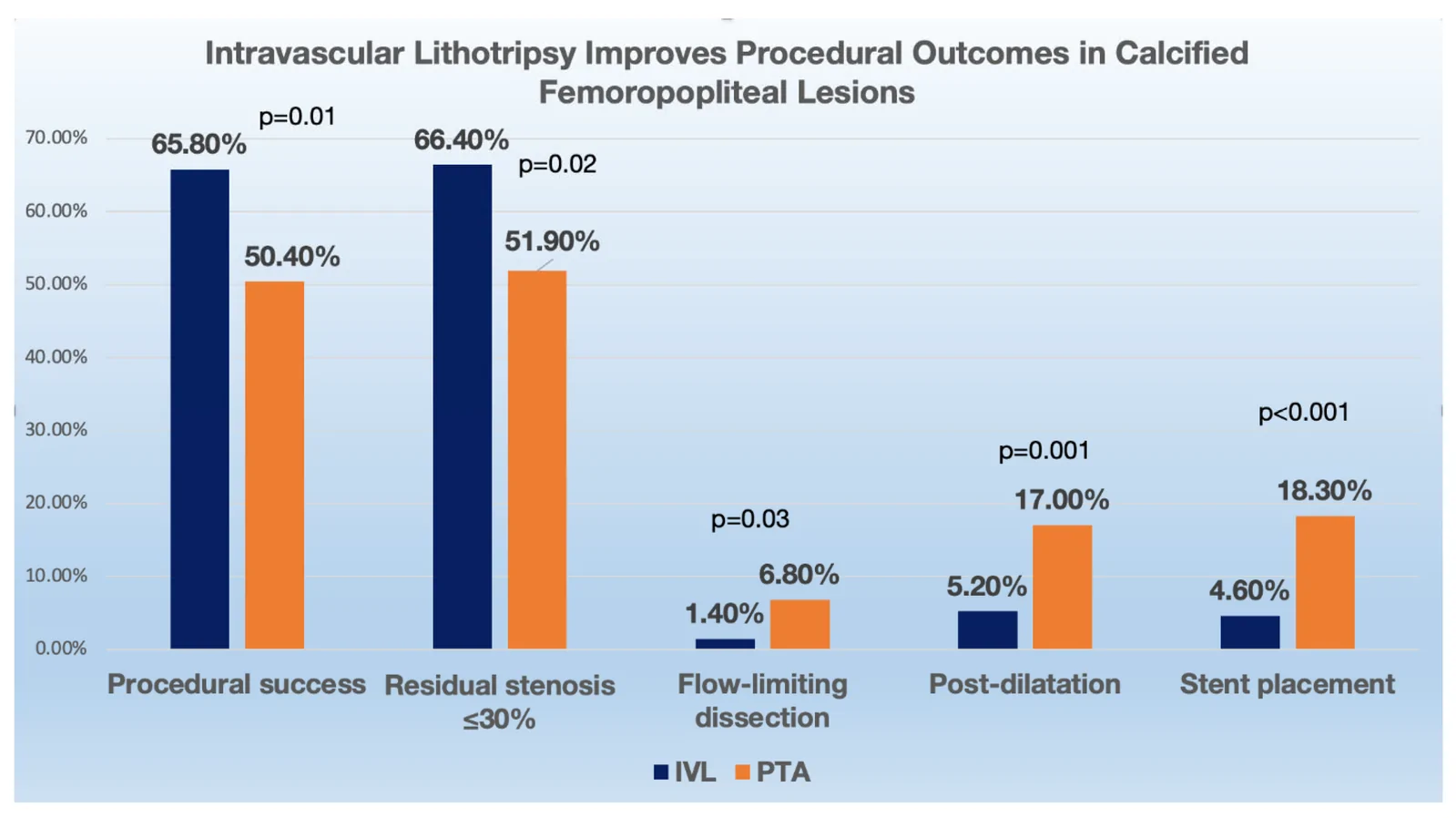
Procedural outcomes after intravascular lithotripsy (IVL) versus percutaneous transluminal angioplasty (PTA) for vessel preparation in calcified femoropopliteal lesions.

Furthermore evidence from peripheral circulation further illustrates the mechanical benefits of lesion preparation, particularly in heavily calcified disease. In a randomized study of 306 patients with moderately to severely calcified femoropopliteal lesions, intravascular lithotripsy (IVL) before DCB treatment or provisional stenting improved acute procedural outcomes compared with conventional percutaneous transluminal angioplasty (PTA). Procedural success was higher with IVL (65.8% vs. 50.4%; *p* = 0.01), while flow-limiting dissection (1.4% vs. 6.8%; *p* = 0.03), post-dilatation (5.2% vs. 17.0%; *p* = 0.001), and provisional stenting (4.6% vs. 18.3%; *p* < 0.001) were all less frequent. Thirty-day major adverse events were uncommon in both groups (0% vs. 1.3%; *p* = 0.16), and clinically driven target lesion revascularization was identical at 0.7% (*p* = 1.00) [13] (Figure 1).

The longer-term results showed higher primary patency with IVL at 1 year (80.5% vs. 68.0%; *p* = 0.017) and 2 years (70.3% vs. 51.3%; *p* = 0.003). However, at 1 year, freedom from CD-TLR (95.7% vs. 98.3%; *p* = 0.94) and freedom from restenosis (90.0% vs. 88.8%; *p* = 0.48) were similar. The patency advantage was therefore driven largely by the lower rate of provisional stenting, which was incorporated into the prespecified primary-patency definition, rather than by demonstrably greater DCB efficacy or drug uptake [13].

Taken together, coronary and peripheral evidence points to different measurable effects of vessel preparation. In coronary DCB treatment, calcium burden and vessel-wall morphology are associated with subsequent lesion failure, while data further show that proximal location, diffuse disease, and prior CABG identify lesions at greater risk of requiring unplanned stenting after DCB-only PCI. Peripheral randomized data more directly demonstrate improved procedural predictability, fewer flow-limiting dissections, and less bailout stenting after calcium modification. Thus, across vascular beds, optimal preparation appears to improve the mechanical conditions required for a successful stentless result; however, whether these improvements directly enhance tissue drug transfer remains incompletely established.

## 3. Contemporary Strategies for Vessel Preparation

The choice of vessel-preparation strategy should be guided by the mechanical problem that must be solved before definitive therapy. In relatively compliant lesions, conventional balloons (semi and non-compliant) may be sufficient to achieve luminal expansion and facilitate DCB delivery. As fibrosis, calcification, lesion length, or mechanical resistance increase, conventional balloon-based preparation may no longer provide predictable expansion, and more targeted plaque modification may become necessary. The practical distinction is therefore between strategies that primarily improve luminal compliance and those that modify plaque sufficiently to overcome a specific mechanical limitation [14].

### 3.1. Balloon-Based Preparation: From Simple Dilatation to Targeted Plaque Modification

Conventional balloon angioplasty remains the foundation of vessel preparation because it is widely available, technically straightforward, and sufficient for many mechanically responsive lesions. Semi-compliant balloons are often suitable for initial dilatation, whereas non-compliant (NC) balloons provide greater radial force and more predictable diameter control at higher inflation pressures, making them useful when additional resistance must be overcome.

Evidence supporting high-pressure NC balloon preparation is derived largely from stent-based PCI and should therefore be interpreted as mechanistic rather than DCB-specific. In the pooled Pre-NC and OPRENBIS randomized studies, comprising 104 patients and 110 lesions, NC balloon predilatation was performed at substantially higher pressures than semi-compliant balloon preparation (24 ± 7 vs. 14 ± 3 atm; *p* < 0.0001). Immediate stent expansion was similar between strategies (stent expansion index [SEI], 0.88 ± 0.13 vs. 0.85 ± 0.14; *p* = 0.18), but after post-dilatation, final expansion was greater in lesions prepared with NC balloons (SEI, 0.94 ± 0.13 vs. 0.88 ± 0.13; *p* = 0.02), without an apparent increase in major procedural complications. Importantly, these studies included relatively uncomplicated lesions and were not powered for clinical outcomes. Their relevance to DCB therapy therefore lies principally in demonstrating that more controlled high-pressure balloon preparation can improve acute mechanical expansion, rather than establishing that more aggressive predilatation improves long-term DCB outcomes [14].

When conventional balloon angioplasty produces incomplete, heterogeneous, unstable expansion or results in excessive recoil, cutting or scoring balloons may provide a more controlled means of lesion modification. By concentrating force along predefined elements of the balloon surface, these devices create focal plaque disruption, reduce balloon slippage, and may improve expansion in fibrotic, fibrocalcific, or restenotic tissue [13,15].

A persistent balloon waist despite appropriate sizing and high-pressure inflation should be regarded as evidence of unresolved mechanical resistance rather than an indication for repeated indiscriminate dilatation. In lesions with thick, concentric, or deep calcium, further balloon inflation may increase vessel-wall injury without producing meaningful additional expansion. Specialty balloons may remain appropriate when controlled focal modification is likely to overcome the residual resistance, whereas atherectomy or intravascular lithotripsy (IVL) should be considered when calcification represents the dominant mechanical limitation. Selection should be guided by lesion crossability, intravascular imaging findings on calcium depth and distribution, and the mechanism responsible for persistent under-expansion [16].

In pooled real-world analyses, preparation with cutting or scoring balloons has been associated with lower residual stenosis after DCB angioplasty compared with conventional balloon preparation [11]. However, whether more intensive balloon-based preparation improves durable clinical outcomes remains uncertain. This question is being explored further in the PICCOLETO XI A-PREP study (NCT07391033), a multicenter international retrospective cohort comparing intensive lesion preparation with cutting, scoring, and/or OPN balloons against standard preparation with semi-compliant or NC balloons before DCB angioplasty for ISR. The primary endpoint is 12-month major adverse cardiovascular events, with additional procedural and angiographic outcomes [17].

### 3.2. Atherectomy

Atherectomy occupies a distinct role in vessel preparation because it directly modifies calcified plaque rather than relying solely on balloon-mediated expansion. Its value lies not simply in the presence of calcium, but in overcoming mechanical limitations that conventional or specialty balloons cannot address, particularly when severe calcification impairs lesion crossability, balloon expansion, or device delivery. Rotational and orbital atherectomy predominantly modify superficial calcium, increasing lesion compliance and facilitating subsequent balloon-based treatment [18].

Most randomized coronary atherectomy evidence is stent-based and therefore indirect for DCB therapy. ROTAXUS and PREPARE-CALC demonstrated improved procedural or strategy success with rotational atherectomy in severely calcified lesions, but without a consistent long-term clinical advantage; randomized orbital atherectomy data similarly showed no significant reduction in 1-year target-vessel failure compared with balloon angioplasty [19,20,21,22]. These studies support atherectomy as a tool for overcoming resistant calcified anatomy and improving procedural deliverability, rather than as evidence that routine atherectomy improves long-term DCB-specific outcomes.

Overall, randomized evidence supports a selective rather than routine role for atherectomy. Its greatest value is in addressing specific mechanical limitations, including lesion uncrossability, impaired device delivery, or persistent under-expansion due to resistant superficial calcium. Severe angiographic calcification alone should not mandate atherectomy when acceptable expansion can be achieved with less complex strategies.

**Figure 2:**
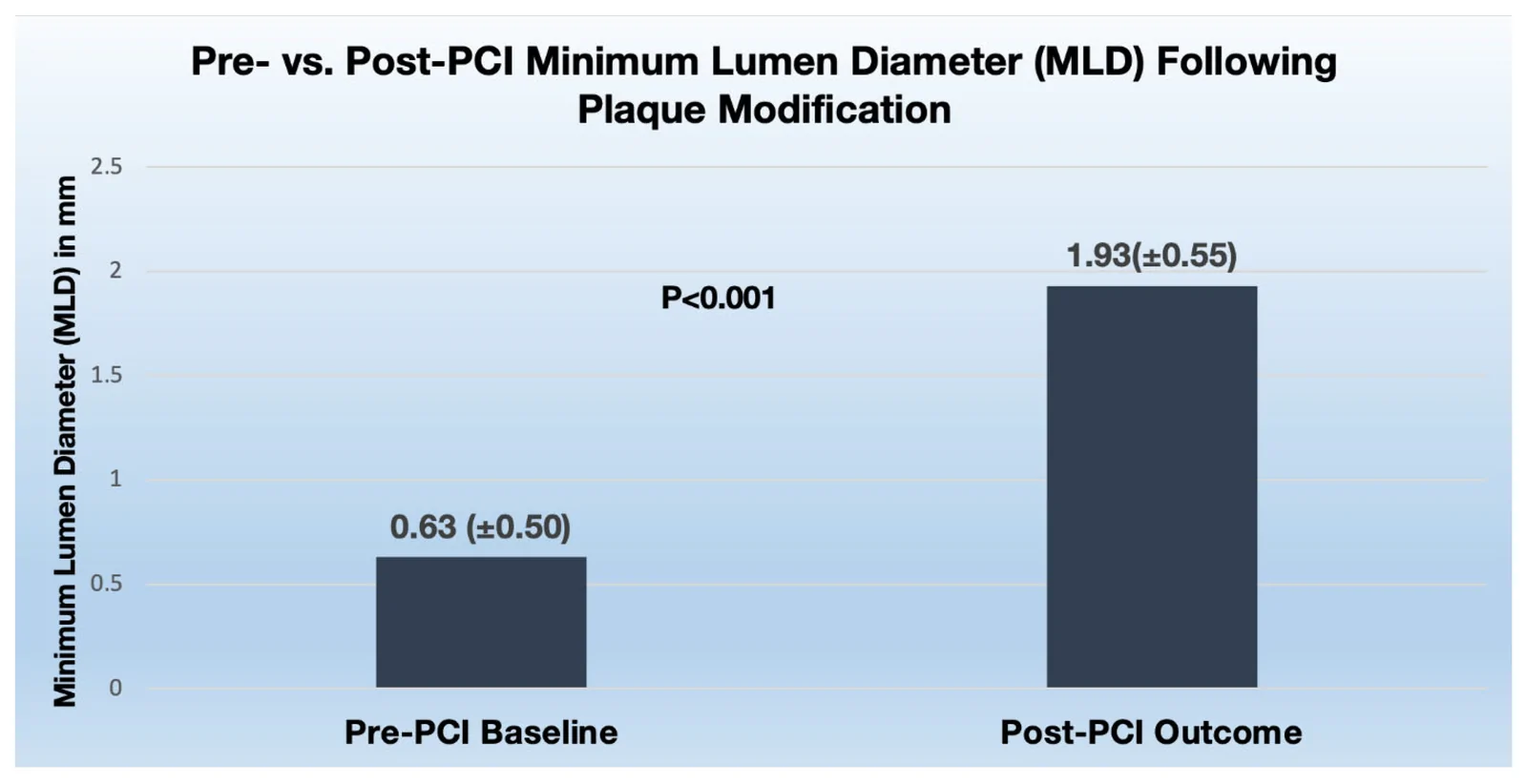
Pre- and Post-Procedural Minimum Lumen Diameter. Changes in minimum lumen diameter (mm) before (Pre-PCI) and after (Post-PCI) plaque modification using a solo-drug coated balloon strategy.

**Figure 3:**
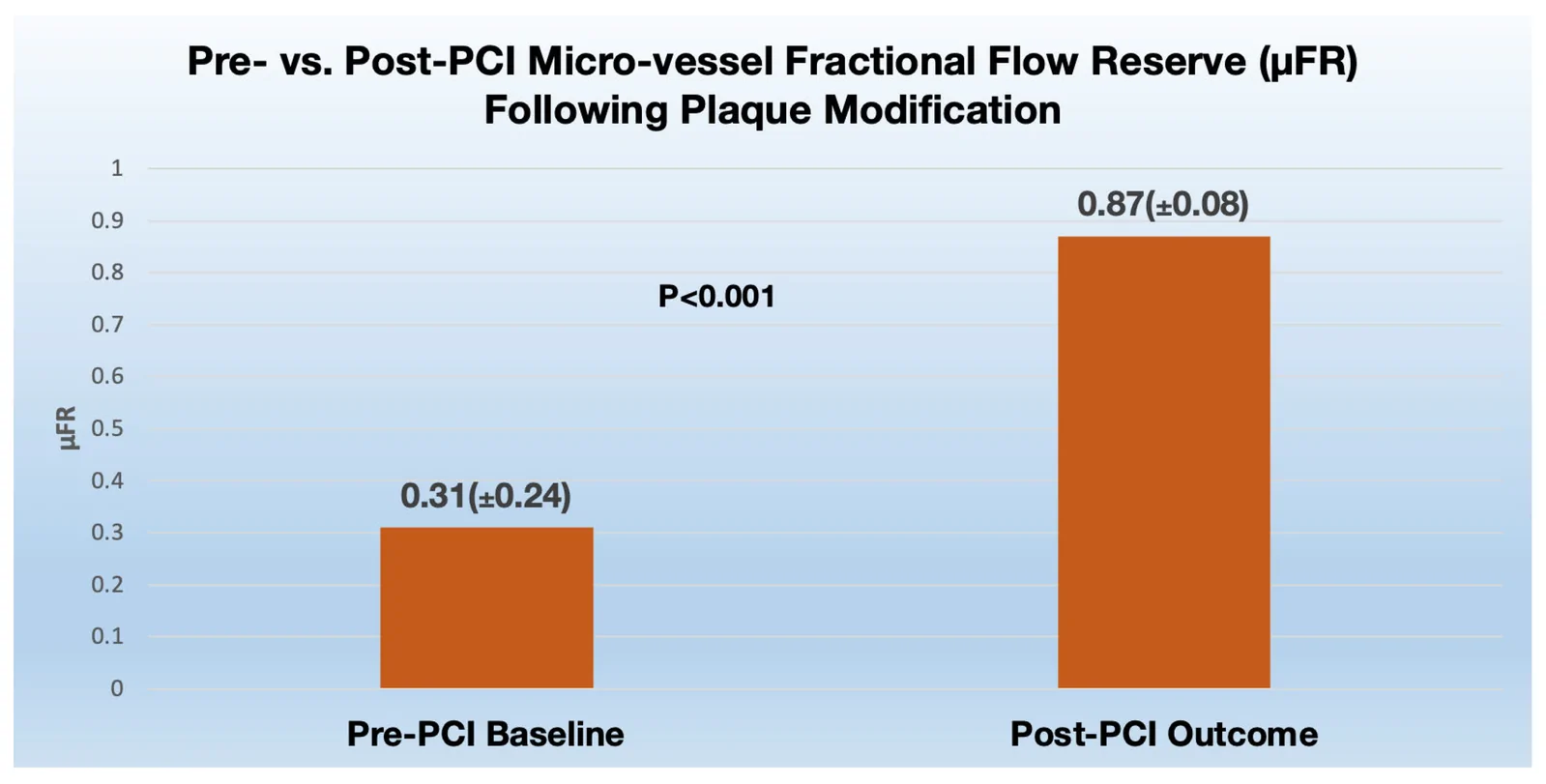
Pre- and Post-Procedural Micro-vessel Fractional Flow Reserve. Changes in micro-vessel fractional flow reserve (μFR) indices before (Pre-PCI) and after (Post-PCI) lesion preparation [23].

**Figure 4:**
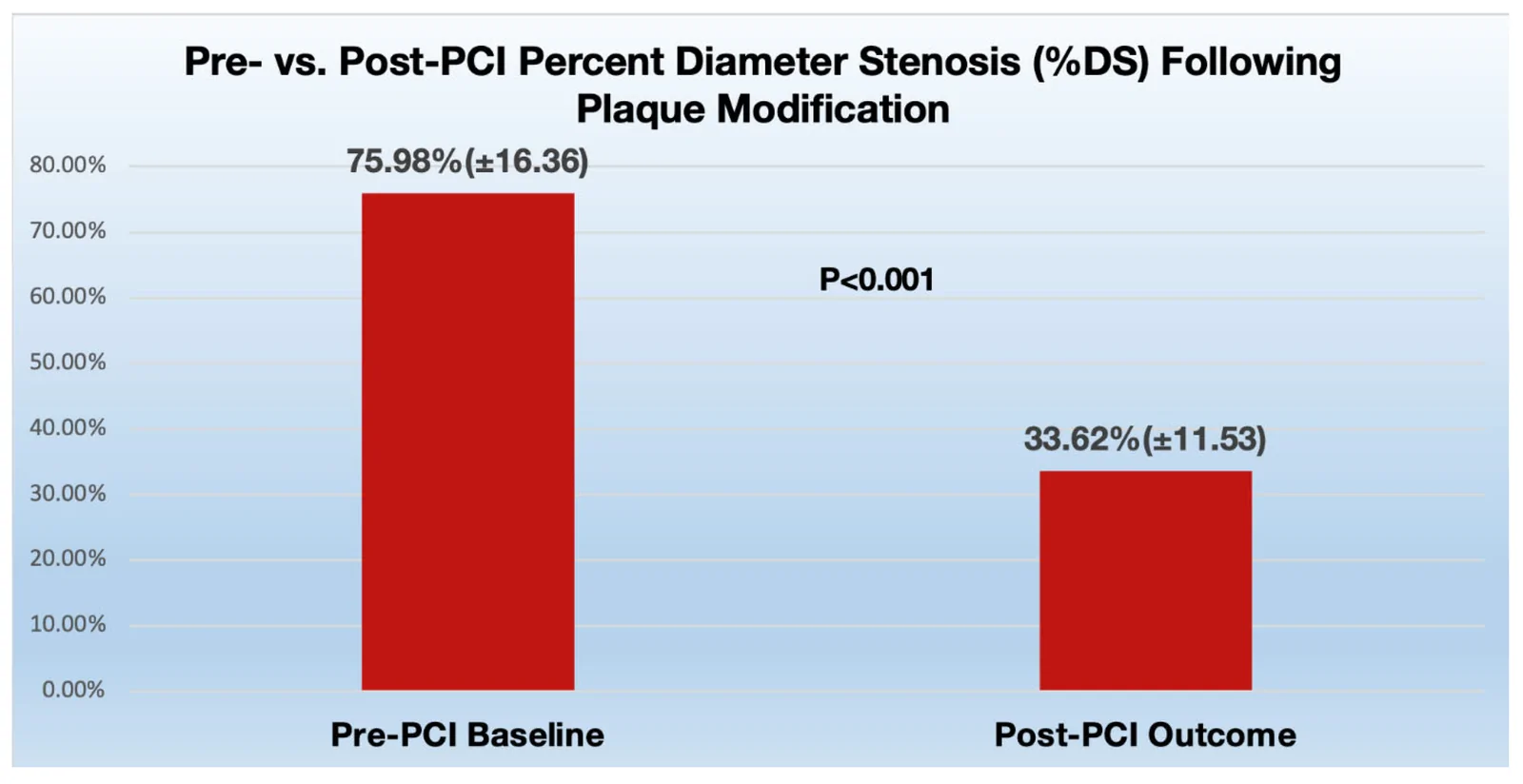
Pre- and Post-Procedural Percent Diameter Stenosis. Changes in percent diameter stenosis (%) before (Pre-PCI) and after (Post-PCI) vessel treatment in calcified coronary lesions (N = 121) [23].

Emerging DCB-specific data nevertheless support the feasibility of atherectomy as part of a scaffold-free strategy in selected calcified lesions. In the CARDIAC study, 121 patients with severely calcified de novo coronary lesions underwent plaque modification followed by DCB-only treatment. IVL was used in 53%, RA in 37%, orbital atherectomy in 17%, and combined atherectomy and IVL in 6%. Procedural success was 100%, while dissection occurred in 21%, predominantly type A or B. At a median follow-up of 384 days, target-lesion failure occurred in 6.7%, including TLR in 5.0% and nonfatal MI in 1.7%, with no cardiac deaths. Minimum lumen diameter increased from 0.63 to 1.93 mm and diameter stenosis decreased from 75.98% to 33.62% [23] (Figure 2, Figure 3 and Figure 4).

These findings support the feasibility of DCB treatment after intensive plaque modification but should be interpreted cautiously. CARDIAC was observational and included several calcium-modification strategies, preventing attribution of outcomes specifically to atherectomy. Its main value is therefore as a bridge between the predominantly stent-based atherectomy literature and emerging DCB-specific practice.

### 3.3. Intravascular Lithotripsy (IVL)

IVL modifies calcified plaque by generating acoustic pressure waves that fracture calcium in situ. Unlike atherectomy, it does not require direct plaque ablation and can affect both superficial and deep calcium. IVL is therefore particularly suited to balloon-crossable lesions with deep, concentric, or circumferential calcification limiting vessel expansion.

In Disrupt CAD III, IVL was evaluated in 431 patients with severely calcified de novo coronary lesions undergoing stent implantation, achieving 92.4% procedural success and 92.2% freedom from 30-day MACE. OCT demonstrated calcium fracture in 67.4% of lesions, confirming effective modification of heavily calcified plaque [24]. Similar procedural safety and efficacy were observed across the pooled Disrupt CAD I–IV studies, with high stent-delivery success and low rates of serious angiographic complications [5].

An important advantage of IVL is its limited dependence on wire bias and ability to modify deep calcium while preserving side-branch access, making it useful in selected complex and bifurcation lesions [5]. However, most Disrupt CAD studies were single-arm and stent-based, demonstrating feasibility and safety rather than superiority over atherectomy or balloon-based preparation.

IVL is also limited by the need for balloon delivery across the lesion. Severely stenotic, balloon-uncrossable, or highly resistant lesions may therefore require atherectomy before IVL. Conversely, when conventional balloons achieve adequate expansion, the incremental value of IVL remains uncertain. Thus, IVL should be selected according to calcium morphology and the residual mechanical limitation rather than used routinely for calcification alone [23].

### 3.4. When Different Preparation Strategies Need to Be Combined

The mechanical limitations of individual technologies have also created interest in sequential or hybrid preparation. A lesion that is balloon-uncrossable may first require atherectomy to create a passage for a balloon, after which IVL can address residual deep or circumferential calcium. Conversely, a lesion that crosses readily but remains resistant to expansion may be more appropriately treated directly with IVL [25,26,27].

Evidence supporting combined strategies remains limited, however, and largely consists of observational experience. Hybrid preparation should therefore be regarded as a lesion-specific strategy rather than a routine escalation pathway.

### 3.5. Vessel Preparation Beyond Calcium: Laser and Directional Atherectomy

Not all lesions requiring modification are dominated by calcium. Fibrotic tissue, thrombotic material, neointimal hyperplasia, and resistant restenotic tissue may require a different approach. Excimer laser coronary atherectomy (ELCA) is therefore best considered a selective rather than routine preparation strategy.

The strongest randomized evidence for ELCA comes from the EXCITE ISR trial, which evaluated femoropopliteal in-stent restenosis. ELCA plus balloon angioplasty improved procedural success compared with balloon angioplasty alone (93.5% vs. 82.7%; *p* = 0.01) and improved 6-month freedom from TLR (73.5% vs. 51.8%; *p* < 0.005) [28].

Evidence more directly relevant to coronary DCB comes from the observational study by Shibui et al., in which ELCA was used before paclitaxel-coated balloon angioplasty in de novo coronary lesions. Because ELCA was preferentially used in more complex lesions, the study provides useful feasibility data [29].

Directional atherectomy provides a more direct example of plaque debulking before DCB therapy in the peripheral circulation. In the DEFINITIVE AR study, directional atherectomy was evaluated for femoropopliteal artery disease. Directional atherectomy followed by DCB improved technical success (89.6% vs. 64.2%; *p* = 0.004) and reduced flow-limiting dissection (2% vs. 19%; *p* = 0.01) compared with DCB alone. However, 1-year angiographic diameter stenosis, target lesion revascularization, and duplex-derived patency were not significantly different between groups [30].

The distinction is important: more effective preparation improved the procedural result, but did not demonstrate superior 1-year clinical or patency outcomes. The ≤30% residual-stenosis threshold was the prespecified definition of technical success rather than a definitive therapeutic target proven to improve DCB durability [30].

The broader evidence is consistent with this interpretation. In the 2025 systematic review and network meta-analysis by Yiu et al., four randomized trials comprising 549 patients evaluated atherectomy or IVL versus plain balloon angioplasty before DCB treatment of femoropopliteal disease. Atherectomy or IVL significantly reduced bailout stenting (RR 0.25; 95% CI, 0.07–0.89), but did not significantly improve freedom from target-lesion reintervention, major amputation, or mortality at 1 year [31]. Thus, across peripheral interventions, advanced vessel preparation appears to improve procedural predictability more consistently than it improves patient-centered long-term outcomes.

### 3.6. Choosing the Preparation Strategy: A Lesion-Based Approach

The available evidence supports a stepwise, lesion-based rather than device-driven approach to vessel preparation. Balloon angioplasty remains appropriate for lesions that are mechanically responsive, with NC, cutting, or scoring balloons providing progressively greater control of plaque modification. Atherectomy becomes more relevant when balloon crossing or expansion is not achievable, particularly when superficial calcium or plaque burden is the dominant mechanical limitation. IVL is particularly suited to balloon-crossable lesions in which deep or circumferential calcium prevents adequate expansion. Laser and directional atherectomy have more selective roles when fibrotic, thrombotic, restenotic, or bulky plaque requires modification, particularly in selected coronary and peripheral lesions [32,33] (Table 1).

Importantly, the evidence across both coronary and peripheral vascular beds does not support the concept that more aggressive preparation is inherently better. The most consistent benefit of advanced preparation is improvement in procedural conditions, greater luminal gain, fewer flow-limiting dissections, reduced bailout stenting, or improved device delivery. Whether these procedural improvements translate into greater drug delivery or more durable DCB-mediated inhibition of restenosis remains less certain [32].

Of note, more intensive lesion preparation is not necessarily preferable and may expose patients to additional procedural risk. Repeated high-pressure balloon inflation or unnecessary escalation to atherectomy or multiple plaque-modification devices may increase the risk of dissection, perforation, distal embolization, or other vessel injury without established additional benefit for long-term DCB outcomes [34]. Accordingly, preparation should be proportional to the dominant mechanical limitation, with escalation reserved for lesions in which simpler balloon-based strategies do not achieve adequate expansion or vessel stability. The objective should be the least intensive strategy capable of creating an adequate treatment substrate rather than maximal plaque modification.

## 4. Clinical Evidence for Vessel Preparation Before DCB Therapy: Comparative Perspective Across Coronary and Peripheral Interventions

The evidence spans three related but distinct questions: whether DCB therapy is effective after conventional angioplasty, whether preparation quality predicts DCB outcomes, and whether advanced preparation technologies improve the treatment substrate.

### 4.1. Coronary Lesion Preparation for DCB Therapy

Konishi et al. provided quantitative evidence linking the quality of lesion preparation to outcomes after coronary DCB therapy. Among 936 patients treated with DCB between 2014 and 2017, 247 underwent DCB-only treatment for de novo lesions, with IVUS available in 188. A pre-DCB residual plaque area ≥ 58.5% was associated with target lesion failure (TLF) (AUC, 0.81) and remained an independent predictor of TLF (HR, 7.59; *p* < 0.01) [9]. These findings suggest that, in the absence of a permanent scaffold, residual plaque burden may be an important determinant of the final treatment substrate.

Randomized evidence from ISAR-DESIRE 4 further supports the importance of controlled preparation in coronary ISR. Among 252 patients with DES-ISR, scoring-balloon predilatation before paclitaxel-coated balloon treatment reduced follow-up diameter stenosis (35.0 ± 16.8% vs. 40.4 ± 21.4%; *p* = 0.047) and binary restenosis (18.5% vs. 32.0%; *p* = 0.026), although target-lesion revascularization was not significantly reduced (16.2% vs. 21.8%; *p* = 0.26) [35]. Thus, more targeted neointimal modification improved angiographic efficacy, but a corresponding clinical benefit was not demonstrated.

### 4.2. Peripheral DCB: From Conventional Angioplasty to Advanced Preparation

IN.PACT SFA and LEVANT 2 established the efficacy of DCB following conventional balloon angioplasty. IN.PACT SFA randomized 331 patients 2:1 to DCB or PTA and demonstrated 12-month primary patency of 82.2% versus 52.4% (*p* < 0.001), with clinically driven TLR of 2.4% versus 20.6% (*p* < 0.001) [36]. LEVANT 2 randomized 476 patients 2:1 and demonstrated 12-month primary patency of 65.2% versus 52.6% (*p* = 0.02), with non-inferior safety [37]. These trials demonstrate the efficacy of antiproliferative drug delivery after conventional balloon preparation but do not establish that atherectomy or IVL is necessary before DCB.

More direct evidence comes from preparation-quality studies. Tomoi et al. retrospectively evaluated 268 patients involving 308 limbs treated with femoropopliteal DCB therapy. Successful preparation was defined as <50% residual stenosis with dissection below grade D. Over a median 4.4-year follow-up, successful preparation was associated with higher 5-year primary patency (51.2% vs. 31.5%; *p* < 0.001), freedom from CD-TLR (65.0% vs. 51.6%; *p* = 0.01), and limb salvage (94.4% vs. 87.9%; *p* = 0.03) [38].

DEFINITIVE AR provides randomized evidence regarding advanced preparation. The trial randomized 102 patients to directional atherectomy plus DCB (n = 48) or DCB alone (n = 54), with an additional 19 severely calcified patients treated with atherectomy plus DCB. Technical success was higher with atherectomy plus DCB (89.6% vs. 64.2%; *p* = 0.004), and flow-limiting dissection was lower (2% vs. 19%; *p* = 0.01). Moreover, 1-year angiographic stenosis (33.6 ± 17.7% vs. 36.4 ± 17.6%; *p* = 0.48), CD-TLR (7.3% vs. 8.0%; *p* = 0.90), duplex patency (84.6% vs. 81.3%; *p* = 0.78), and freedom from major adverse events (89.3% vs. 90.0%; *p* = 0.86) were comparable [30].

**Table 1:** Key evidence on vessel preparation before DCB Therapy.

Study	Vascular Bed/Population	Preparation Strategy	Key Findings	Study Design/Level of Evidence	Clinical Interpretation
Konishi et al. [9]	Coronary DCB; 247 de novo DCB-only lesions	IVUS-guided assessment of residual plaque	Residual plaque area ≥ 58.5% predicted TLF (AUC, 0.81; HR, 7.59; *p* < 0.01)	Observational cohort study	Supports residual plaque burden as a potential imaging marker of an unfavorable DCB treatment substrate and subsequent TLF; observational evidence does not establish a treatment threshold.
ISAR-DESIRE 4 [35]	Coronary DES-ISR; 252 patients	Scoring-balloon vs. conventional balloon predilatation before PCB	Lower diameter stenosis (35.0% vs. 40.4%; *p* = 0.047) and binary restenosis (18.5% vs. 32.0%; *p* = 0.026), but no significant difference in TLR	Randomized controlled trial	Controlled preparation improves angiographic outcomes without proven clinical benefit
Tomoi et al. [38]	Femoropopliteal DCB; 268 patients/308 limbs	Successful preparation: <50% residual stenosis and <grade D dissection	5-year primary patency 51.2% vs. 31.5%, CD-TLR-free survival 65.0% vs. 51.6%, limb salvage 94.4% vs. 87.9%	Retrospective cohort study	Supports an association between preparation quality and long-term outcomes; retrospective design limits causal inference
DEFINITIVE AR [30]	Femoropopliteal DCB; 102 randomized patients	Directional atherectomy + DCB vs. DCB alone	Higher technical success (89.6% vs. 64.2%; *p* = 0.004) and less flow-limiting dissection (2% vs. 19%; *p* = 0.01); no significant 1-year difference in stenosis, CD-TLR, or patency	Multicenter randomized controlled pilot trial	Advanced preparation improves procedural outcomes but has not demonstrated consistent long-term superiority
Disrupt PAD III [4]	Calcified femoropopliteal lesions; 306 patients	IVL vs. PTA before DCB or provisional stenting	Higher procedural success (65.8% vs. 50.4%; *p* = 0.01), less flow-limiting dissection (1.4% vs. 6.8%; *p* = 0.03), and less provisional stenting (4.6% vs. 18.3%; *p* < 0.001); primary patency 80.5% vs. 68.0% at 1 year and 70.3% vs. 51.3% at 2 years	Prospective multicenter randomized controlled trial	Strong evidence for improved procedural performance; patency findings require cautious interpretation because provisional stenting was incorporated into the endpoint
Yiu et al. [31]	Peripheral DCB; 4 RCTs, 549 patients	Atherectomy/IVL vs. PTA before DCB	Advanced preparation reduced bailout stenting (RR, 0.25; 95% CI, 0.07–0.89) but did not significantly improve 1-year target-lesion reintervention, major amputation, or mortality	Systematic review and network meta-analysis of randomized controlled trials	Advanced preparation provides a predominantly procedural advantage; long-term DCB-specific benefit remains uncertain

Abbreviations: CD-TLR, clinically driven target-lesion revascularization; DCB, drug-coated balloon; DES, drug-eluting stent; IVL, intravascular lithotripsy; IVUS, intravascular ultrasound; LLL, late lumen loss; MLD, minimum lumen diameter; PCB, paclitaxel-coated balloon; PTA, percutaneous transluminal angioplasty; TLF, target-lesion failure; TVF, target-vessel failure.

**Figure 5:**
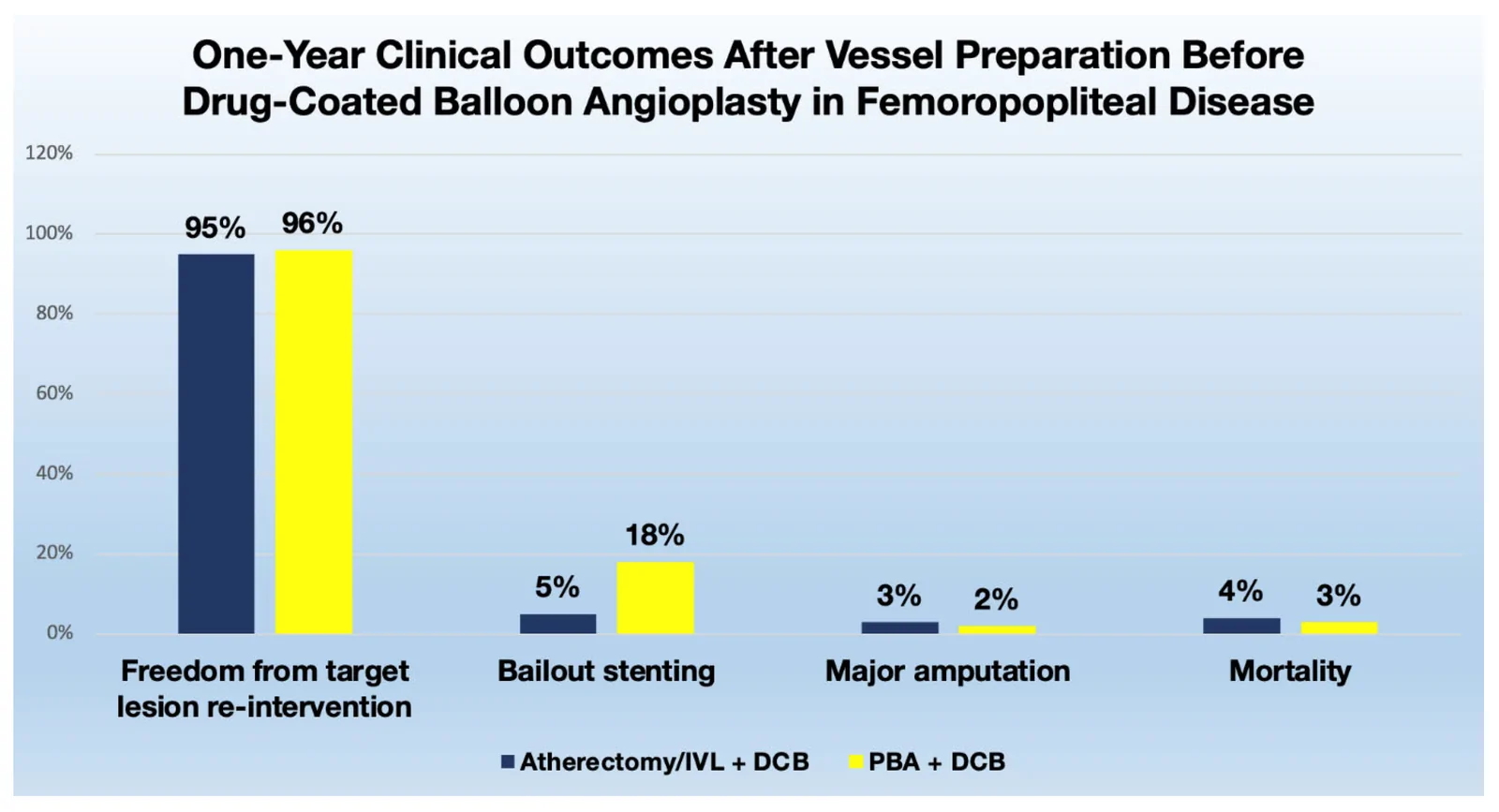
Comparative one-year outcomes of atherectomy or intravascular lithotripsy (IVL) plus drug-coated balloon (DCB) versus plain balloon angioplasty (PBA) plus DCB in patients with femoropopliteal atherosclerotic disease. Reported relative effects are presented as risk ratios (RRs) with 95% confidence intervals (CIs): freedom from target lesion re-intervention, RR 0.99 (95% CI 0.95–1.02); bailout stenting, RR 0.25 (95% CI 0.07–0.89); major amputation, RR 1.04 (95% CI 0.13–8.58); and mortality, RR 1.25 (95% CI 0.48–3.27). Absolute event rates were 95% (91–98%) vs. 96% for freedom from target lesion re-intervention, 5% (1–16%) vs. 18% for bailout stenting, 3% (1–17%) vs. 2% for major amputation, and 4% (1–10%) vs. 3% for mortality, respectively.

The 2025 systematic review and network meta-analysis by Yiu et al. compared plain balloon angioplasty, atherectomy, and IVL before femoropopliteal DCB therapy. The analysis focused on one-year freedom from target-lesion reintervention and secondary outcomes including bailout stenting, major amputation, and mortality. Overall, advanced preparation reduced bailout stenting but did not demonstrate a clear significant improvement in one-year freedom from target-lesion reintervention [31]. These findings support a procedural rather than consistently demonstrated long-term advantage of advanced preparation (Figure 5).

### 4.3. Coronary Versus Peripheral Disease: Different Targets, Same Principle

Across vascular beds, the central principle is similar: DCB therapy requires conversion of the lesion into a predictable treatment substrate, but the practical definition of adequate preparation differs. Coronary studies increasingly quantify residual plaque burden and lumen dimensions, whereas peripheral studies more commonly use residual stenosis and dissection grade. These differences reflect vessel size, plaque morphology, biomechanics, lesion length, imaging adoption, and clinical endpoints [39].

Accordingly, preparation should be vessel- and lesion-centered rather than device-centered. A relatively compliant lesion may require controlled balloon expansion, whereas resistant superficial calcium may require scoring/cutting balloons or atherectomy, and balloon-crossable deep or circumferential calcium may favor IVL. The objective is sufficient modification to overcome the dominant mechanical limitation while minimizing unnecessary vessel injury.

### 4.4. Calcium Modification in DCB Therapy vs. Stent Strategy

DCB-specific evidence remains limited but is emerging. In the prospective BENELUX-IVL registry, 45 of 579 patients treated with IVL subsequently received DCB and 534 received DES. Procedural success was similar (89.4% vs. 91.7%; *p* = 0.55), with no bailout DES required in the DCB group; in-hospital MACE was 0% vs. 2.1% (*p* = 0.33). DCB use was more frequent in ISR (60.0% vs. 29.6%; *p* < 0.01) and CTO lesions (17.8% vs. 6.8%; *p* < 0.01), supporting a potential “crack-and-drug” strategy in selected calcified lesions [40].

Adequate calcium modification may be particularly relevant to DCB therapy because lesion compliance and balloon–wall contact may influence drug delivery. Intravascular imaging should therefore characterize calcium burden and morphology and guide escalation. In the 2026 ShortCUT IVUS substudy, IVL produced greater MSA than cutting balloons in patients without planned atherectomy with nodular calcium 9.6 ± 3.0 mm^2^ vs. 7.6 ± 1.5 mm^2^ (*p* = 0.04) and 360° calcium (8.9 ± 2.2 mm^2^ vs. 7.4 ± 1.8 mm^2^; *p* = 0.001), although procedural costs were higher. Conversely, after planned rotational atherectomy, cutting balloon and IVL strategies produced comparable results [41].

IVL may increase vessel compliance by modifying calcium in situ, thereby facilitating lumen expansion and more uniform balloon–vessel wall contact. Whether this translates into increased tissue drug transfer during subsequent DCB treatment remains unproven. For DCB, the key endpoint should therefore be sufficient, imaging-confirmed lesion modification rather than selection of a particular calcium-modification device, with escalation determined by calcium morphology and residual mechanical resistance. The BENELUX-IVL data support the feasibility of this concept, but the small DCB cohort and observational design preclude definitive comparisons with DES [42].

### 4.5. ISR: Preparation as Correction of the Failure Mechanism

ISR differs fundamentally from de novo disease because restenosis is often due to mechanical issues with the stents; stent under-expansion from poor lesion preparation, malapposition, and under-sized stents. In addition, residual neointimal tissue, calcific neoatherosclerosis also contribute to ISR. The main stay of ISR treatment is correcting the underlying mechanical causes and for this use of intravascular imaging is the key. Once lesion preparation is complete, routine use of scoring or cutting balloon enhances drug uptake by causing fractures in the hyperplastic tissue. ISAR-DESIRE 4 provides direct randomized evidence that scoring-balloon predilatation can improve the angiographic efficacy of DCB therapy, with lower diameter stenosis (35.0 ± 16.8% vs. 40.4 ± 21.4%; *p* = 0.047) and binary restenosis (18.5% vs. 32.0%; *p* = 0.026) but no significant difference in target lesion revascularization (TLR) (16.8% vs. 22.6%; *p* = 0.25) [35]. Therefore, preparation should correct the dominant mechanism of ISR rather than simply increase treatment intensity. A DCB provides antiproliferative therapy but cannot compensate for severe mechanical obstruction or persistent stent underexpansion.

### 4.6. Assessing Treatment-Substrate Readiness: Recoil, Dissection, and Strategy Conversion

The decision to proceed with DCB should be based on the behavior of the vessel after preparation rather than on balloon expansion during inflation alone. A lesion may appear to expand adequately while the balloon is inflated yet lose a substantial proportion of that gain after deflation. Acute recoil therefore functions as a practical marker of unresolved mechanical resistance. Contemporary coronary DCB practice commonly uses residual stenosis or recoil around 30% as a pragmatic benchmark, but this threshold should be regarded as a decision aid rather than a validated biological boundary. Persistent recoil should prompt reassessment of the underlying substrate and, when appropriate, additional lesion modification or conversion to a scaffold-based strategy [32].

Dissection requires similarly contextual interpretation. Limited vessel-wall injury with preserved TIMI 3 flow and no major true-lumen compromise may be compatible with DCB treatment and can heal during follow-up. Conversely, impaired flow, persistent contrast retention, substantial luminal compression, or an intramural hematoma that compromises the true lumen indicates an unstable substrate and may require further treatment or stent implantation. Intravascular imaging can clarify the depth and extent of injury when angiography is equivocal. Observational data suggesting that some medial injury may coexist with favorable DCB outcomes should not be interpreted as a rationale for deliberately creating deep dissection.

Terminology also matters. Planned crossover to a drug-eluting stent should describe abandonment of the intended DCB strategy because acceptable preparation criteria have not been achieved. Bailout stenting should be reserved for situation where scaffolding is required after DCB treatment either due to recoil or flow limiting dissection. Separating these outcomes is important clinically and in trials because they reflect different mechanisms of DCB-strategy failure.

Taken together, angiographic acceptability and mechanical stability are related but not identical. The aim is not an angiographically perfect vessel; it is a vessel with sufficient lumen, preserved flow, controlled injury, and a corrected dominant mechanical constraint so that permanent scaffolding is not required.

### 4.7. Limitations of Current Evidence

The evidence remains heterogeneous and lacks a common definition of successful preparation. Coronary studies may use residual plaque burden, minimum lumen area, calcium modification, or stent expansion, whereas peripheral studies more often use residual stenosis and dissection grade. In addition, many calcium-modification trials, including PREPARE-CALC, ISAR-CALC, COPS, ROTAXUS, ROTA.shock, and BALI, evaluated stent-based PCI and therefore cannot establish the optimal preparation strategy for DCB-only therapy. Conversely, IN.PACT SFA and LEVANT 2 established DCB efficacy after conventional angioplasty but were not designed to test advanced preparation [19,20,36,37,43].

The available randomized trials are also frequently small and powered primarily for procedural or angiographic endpoints. Improvements in lumen gain, stent expansion, technical success, or dissection do not consistently translate into reductions in TLR, restenosis, or target-vessel failure. Evidence is particularly limited for long diffuse lesions, severe calcification, bifurcations, CTOs, and mixed plaque phenotypes. Future trials should therefore incorporate standardized imaging, objective preparation endpoints, and lesion-specific comparisons to determine whether achieving a predefined preparation target improves drug delivery and durable clinical outcomes.

Ultimately, a lesion should not be considered adequately prepared because a particular device has been used; it should be considered prepared when the resulting vessel geometry and tissue characteristics are sufficient for the intended DCB therapy. This vessel-centered approach may provide the foundation for precision preparation algorithms integrating lesion morphology, calcium distribution, mechanical response, intravascular imaging, and the intended coronary or peripheral DCB application.

## 5. Role and Timing of Intravascular Imaging in DCB Strategy?

The optimal timing of intravascular imaging (IVI) during DCB angioplasty remains unresolved. Unlike DES implantation, where post-procedural imaging primarily confirms stent expansion and detects mechanical complications, DCB therapy depends on adequate lesion preparation without permanent scaffolding. Therefore, imaging performed during lesion assessment and before definitive drug delivery may have greater potential to guide treatment, whereas post-DCB imaging primarily provides mechanistic information on vessel injury, healing, and remodeling.

The randomized ULTIMATE III trial prospectively evaluated IVUS-guided versus angiography-guided DCB angioplasty in 260 high-bleeding-risk patients with de novo coronary lesions (reference vessel diameter 2.0–4.0 mm; lesion length ≤ 15 mm), randomized equally to IVUS-guided (n = 130) or angiography-guided (n = 130) treatment. At 7 months, IVUS guidance significantly reduced in-segment late lumen loss (−0.10 ± 0.34 vs. 0.03 ± 0.52 mm; mean difference 0.14 mm; 95% CI 0.02–0.26; *p* = 0.025), with a larger minimal lumen diameter (2.06 ± 0.62 vs. 1.75 ± 0.63 mm; *p* < 0.001) and lower diameter stenosis (28.15% ± 13.88% vs. 35.83% ± 17.69%; *p* = 0.001). Bailout stenting occurred in 7 versus 2 patients (*p* = 0.172), while target-vessel failure occurred in 1 (0.8%) versus 4 (3.1%) patients (*p* = 0.370) [44].

Additional mechanistic evidence comes from the BENELUX-IVL analysis by Oliveri et al. [42] Although this study evaluated IVL followed by stenting rather than DCB therapy, it provides relevant evidence regarding imaging-based assessment of calcium modification. In a dedicated IVUS substudy of the BENELUX-IVL prospective registry, coronary artery compliance (CACom) was assessed before and after IVL, and the change in compliance (ΔCACom) was evaluated in relation to subsequent stent expansion. ΔCACom was associated with stent expansion, supporting the concept that imaging-derived changes in vessel mechanics may characterize the response to calcium modification [42] However, this was a small, stent-based mechanistic study and therefore cannot be considered a validated imaging endpoint for DCB preparation.

Post-DCB imaging provides a complementary perspective. In TRANSFORM I, 121 patients with de novo small-vessel coronary disease were randomized to sirolimus-coated balloon or paclitaxel-coated balloon angioplasty [45] The trial used OCT to guide appropriate DCB sizing, while 109 patients contributed to the angiographic analysis. SCB did not demonstrate noninferiority to PCB for 6-month angiographic net lumen gain, while OCT provided information regarding acute vessel injury and subsequent lumen changes [45]. Beyond its role in sizing, OCT enabled quantitative assessment of post-procedural dissection and tissue composition at the site of dissection; in 100 lesions from 98 patients, dissection was identified in 96% (96/100) of lesions, with a median dissection volume of 1.98 mm^3^ and a median maximal dissection arc of 113° [46]. In a serial OCT substudy, 19 patients with 21 lesions were analyzed (SCB: 9 patients/11 lesions; PCB: 10 patients/10 lesions). In the SCB group, lumen volume decreased from 97.35 ± 71.09 mm^3^ immediately after treatment to 87.96 ± 61.48 mm^3^ at 6 months (*p* = 0.03), whereas no significant change was observed in the PCB group (69.67 ± 38.24 mm^3^ vs. 71.64 ± 42.22 mm^3^; *p* = 0.64). Late lumen volume loss was 9.39 ± 12.76 mm^3^ with SCB versus −1.97 ± 12.90 mm^3^ with PCB (*p* = 0.06). The relationship between dissection volume and late lumen volume loss showed a trend toward interaction between treatment groups (*p* = 0.07) [47]. Thus, OCT can complement angiography by providing quantitative lesion-level assessment of post-procedural dissection and subsequent luminal changes after DCB angioplasty.

Taken together, the available evidence suggests that IVI performed during lesion assessment and preparation before definitive DCB therapy has greater potential to influence treatment decisions than post-DCB imaging, although an optimal imaging time point or universal imaging-defined preparation threshold has not yet been established. TRANSFORM I, in contrast, illustrates the value of OCT for characterizing vessel injury and subsequent remodeling rather than demonstrating a strategy-changing role for post-DCB imaging [42,44,45].

Emerging ultrasound techniques may provide additional information on vascular microstructure and subclinical vascular changes. Deep-learning-based super-resolution ultrasound and ultrasound localization microscopy can improve visualization of small vessels and microvascular architecture beyond conventional ultrasound resolution, while recent contrast-free approaches may facilitate faster microvascular assessment [48,49]. However, these techniques remain investigational, with important limitations related to validation, acquisition, contrast-agent dependence, and clinical translation. Their potential role in characterizing vascular injury or treatment response after vessel preparation is therefore promising but remains to be established.

Future studies should establish standardized pre-DCB IVI criteria incorporating lumen expansion, residual plaque burden, recoil, calcium modification, and acceptable dissection. Such imaging-defined targets could support lesion-specific escalation of preparation and help identify lesions suitable for a leave-nothing-behind DCB strategy versus those requiring additional intervention or DES implantation.

## 6. Practical Algorithm for Vessel Preparation in Peripheral Artery Disease

No universally validated imaging-defined threshold defines a “DCB-ready” vessel. Accordingly, post-preparation assessment should integrate angiographic, mechanical, and, when available, intravascular imaging findings. The practical objective is sufficient lumen, preserved flow, controlled vessel-wall injury, and correction of the dominant mechanical constraint, rather than maximal lesion modification. Table 2 summarizes parameters that may inform this assessment and distinguishes pragmatic or observational markers from validated treatment thresholds.

### 6.1. Peripheral DCB Therapy: Characterize → Prepare → Reassess → Treat

Successful peripheral DCB angioplasty requires adequate lesion preparation to maximize luminal expansion, minimize recoil and flow-limiting dissection, and decrease bailout stenting. A modern femoropopliteal algorithm separates treatment into 3 successive steps: (1) lesion characterization, (2) lesion-specific vascular preparation, and (3) definitive treatment based on vessel response after preparation [50,51].

Step 1: Characterize the lesion.

Assess lesion length, calcification, chronic total occlusion (CTO), thrombotic burden, in-stent restenosis (ISR), vessel diameter, and plaque morphology using Duplex ultrasound, computed tomography angiography when appropriate, and IVUS when additional anatomic definition is required. IVUS can improve vessel sizing and characterize plaque and calcium. In a randomized trial of 150 femoropopliteal interventions, IVUS changed the treatment strategy in 79% of cases and increased 12-month freedom from binary restenosis from 55.4% to 72.4% (*p* = 0.008); the benefit was particularly evident among DCB-treated lesions (9.1% vs. 37.5% restenosis; *p* = 0.001) [52].

Step 2: Select preparation according to lesion mechanics.

Short, nonseverely calcified lesions → POBA. 

Plain old balloon angioplasty (POBA) remains the preferred initial preparation when adequate expansion can be achieved without significant recoil or flow-limiting dissection [51].

Fibrotic, moderately calcified, or nondilatable lesions → specialty balloons.

Scoring or cutting balloons may provide controlled plaque modification when conventional balloon expansion is inadequate. However, evidence remains limited, supporting selective rather than routine use [51].

Heavily calcified lesions → IVL or atherectomy.

In Disrupt PAD III, IVL before definitive therapy improved procedural success compared with balloon angioplasty (65.8% vs. 50.4%; *p* = 0.01) and reduced severe dissections (1.4% vs. 6.8%; *p* = 0.03). In the longer-term analysis, primary patency was 80.5% vs. 68.0% at 1 year (*p* = 0.017) and 70.3% vs. 51.3% at 2 years (*p* = 0.003) [13].

Severely fibrotic/calcified or nondilatable lesions → atherectomy.

Atherectomy may be an option when plaque debulking is needed, particularly in cases of extremely calcified or nondilatable lesions and when permanent implantation is not desired. In DEFINITIVE AR, directional atherectomy plus DCB was associated with improved technical success (89.6% vs. 64.2%; *p* = 0.004) and flow-limiting dissection (2% vs. 19%; *p* = 0.01), but 1-year angiographic stenosis (33.6% vs. 36.4%; *p* = 0.48), CD-TLR (7.3% vs. 8.0%; *p* = 0.90), and duplex patency (84.6% vs. 81.3%; *p* = 0.78) were similar. Bailout stenting was uncommon overall (3.7%). Embolization remains an important consideration [30]. A Cochrane review found that atherectomy may reduce dissection (RR 0.28; 95% CI 0.14–0.54) and bailout stenting (RR 0.26; 95% CI 0.09–0.74), but found no clear improvement in primary patency or target-vessel outcomes; the certainty of evidence was very low [53].

Step 3: Reassess and proceed to definitive therapy.

After preparation, reassess luminal expansion, residual stenosis, recoil, dissection, and overall vessel response. Proceed with DCB when an adequate treatment substrate has been achieved; persistent mechanical obstruction or flow-limiting dissection should prompt additional lesion modification or, when necessary, bailout stenting [51]. Thus, preparation should be lesion- and patient-specific rather than device-driven, with the objective of achieving sufficient modification without unnecessary vessel injury.

### 6.2. Coronary DCB Therapy: Characterize → Prepare → Reassess → Treat

Coronary DCB treatment should follow a Characterize → Prepare → Reassess → Treat approach [11].

Step 1: Characterize the lesion.

Assess lesion morphology, plaque burden, calcification, vessel dimensions, and, in ISR, stent expansion and mechanical abnormalities. IVUS or OCT may be used when angiography is insufficient [11].

Step 2: Prepare according to the dominant mechanical limitation.

Conventional balloons may be sufficient for compliant lesions, whereas scoring/cutting balloons, atherectomy, or IVL can be considered for resistant fibrotic or calcified lesions according to lesion morphology and balloon response.

Step 3: Reassess before DCB.

Assess residual stenosis, recoil, dissection, flow, and overall vessel expansion. Proceed with DCB when adequate expansion and stable flow have been achieved without significant flow-limiting injury. Persistent mechanical obstruction or flow-limiting dissection should prompt further modification or, when necessary, conversion to stenting [11].

No universally validated imaging-defined threshold for a “DCB-ready” vessel has yet been established.

## 7. Authors’ Perspective and Future Directions: A Comparative View of Vessel Preparation Before DCB Therapy

From the authors’ perspective, vessel preparation should be regarded as a means of creating an adequate treatment substrate rather than an endpoint in itself. Across coronary and peripheral interventions, the objective is not maximal plaque disruption, but sufficient luminal expansion, controlled vessel injury, and stable balloon–vessel wall contact to facilitate DCB treatment while avoiding unnecessary escalation.

Although this principle is shared across vascular beds, the definition and assessment of adequate preparation differ. In coronary DCB therapy, residual plaque burden, calcium morphology, vessel dimensions, recoil, and dissection can be assessed with intravascular imaging and may provide information beyond angiography. In peripheral disease, particularly femoropopliteal intervention, residual stenosis, recoil, and flow-limiting dissection remain the principal practical measures of procedural adequacy. These differences reflect distinct vessel biomechanics, lesion characteristics, and the current evidence base.

Available evidence does not support routine superiority of advanced preparation over balloon angioplasty; rather, technology selection should be guided by the specific mechanical limitation that persists after initial preparation, rather than lesion complexity alone.

The comparative evidence also highlights the distinction between procedural optimization and demonstrated clinical benefit. In coronary disease, atherectomy and IVL can improve procedural success, calcium modification, lumen dimensions, or stent expansion, but randomized trials have generally not demonstrated consistent reductions in long-term clinical events. Similarly, in peripheral DCB therapy, directional atherectomy and IVL improve technical outcomes and reduce flow-limiting dissection or bailout stenting, whereas randomized evidence has not consistently demonstrated superior long-term target-lesion outcomes. The available network meta-analysis further supports this distinction, showing reduced bailout stenting with advanced preparation without a clear improvement in 1-year freedom from target-lesion reintervention. Procedural improvement should therefore not be equated automatically with greater DCB efficacy.

The importance of the final vessel substrate is nevertheless supported by DCB-specific evidence. In coronary disease, residual plaque burden has been associated with subsequent target-lesion failure after de novo DCB treatment, while scoring-balloon preparation in DES-ISR improves angiographic restenosis without a demonstrated reduction in TLR. In peripheral disease, successful femoropopliteal preparation has been associated with improved long-term patency and limb-related outcomes, although these observational associations do not establish causality. Collectively, these findings suggest that preparation should correct the dominant mechanism of treatment failure, whether residual plaque, neointimal tissue, underexpansion, calcification, recoil, or dissection, rather than simply increase treatment intensity.

We therefore favor a vessel-centered, lesion-specific algorithm:

Characterize → Prepare → Reassess → Treat.

The coronary and peripheral evidence therefore converge on a common principle but with different practical priorities. Coronary DCB therapy may increasingly benefit from imaging-defined assessment of plaque burden and vessel-wall characteristics, whereas peripheral DCB therapy currently relies more heavily on angiographic and mechanical endpoints. Across both territories, the target should be sufficient, not maximal, lesion modification.

Future research should move beyond simple device-to-device comparisons and determine whether achieving a predefined, objectively measured preparation target improves DCB-specific outcomes. Trials should integrate intravascular imaging, calcium characterization, lesion morphology, and procedural response, while evaluating outcomes beyond technical success, including target-lesion failure, restenosis, reintervention, vessel healing, and durable patency. Importantly, studies should distinguish improvements in the mechanical treatment substrate from the still-unproven effect of advanced preparation on tissue drug delivery.

## 8. Conclusions

DCB therapy in both coronary and peripheral disease is facilitated by achieving a predictable treatment substrate rather than maximal lesion modification. Thus, vessel preparation should be lesion and vessel specific, overcoming the primary mechanical constraint and enabling appropriate expansion, controlled injury, and effective balloon–vessel contact. The longevity of DCB in coronary disease may be influenced by residual plaque burden, calcium morphology, and vessel-wall features, while residual stenosis, recoil, and flow-limiting dissection are more closely related to peripheral results. Atherectomy and IVL may enhance procedural success and lesion reduction but their incremental influence on long-term DCB-specific outcomes is unknown. Future trials should establish objective, imaging-based preparation targets and determine whether achieving a “DCB-ready” vessel improves drug delivery and durable clinical outcomes. Ultimately, the goal is not to select the most advanced preparation device, but to identify the least aggressive strategy that reliably prepares the vessel for successful DCB therapy.

Central Illustration. Lesion-specific vessel preparation before drug-coated balloon therapy. A vessel-centered approach integrates lesion characterization, targeted preparation, reassessment, and DCB treatment. Advanced preparation provides more consistent procedural benefits, whereas long-term clinical benefits remain less consistent.

## Figures and Tables

**Table 2:** Practical parameters for assessing DCB readiness after lesion preparation.

Parameter	Coronary DCB Relevance	Peripheral DCB Relevance	Evidence Status/Interpretation
Residual stenosis/recoil	Residual stenosis or recoil around 30% is commonly used as a pragmatic decision aid; persistent recoil suggests unresolved mechanical resistance.	Residual stenosis and recoil remain practical measures of procedural adequacy before DCB.	Pragmatic angiographic/mechanical criteria; no validated biological cutoff.
Flow and vessel stability	Preserved TIMI 3 flow without major true-lumen compromise supports proceeding with DCB.	Stable flow without flow-limiting dissection supports proceeding with DCB.	Clinical/angiographic safety criterion.
Dissection/intramural hematoma	Limited injury with preserved flow may be compatible with DCB; flow impairment, major luminal compression, or a compromising intramural hematoma indicates an unstable substrate.	Flow-limiting dissection remains an important reason for further treatment or bailout stenting.	Contextual criterion; no universal imaging-defined threshold.
Residual plaque burden	IVUS-measured residual plaque burden has been associated with subsequent TLF after de novo coronary DCB treatment.	A corresponding validated peripheral imaging threshold has not been established.	Observational association; not a validated treatment target.
Calcium morphology/modification	IVUS or OCT can characterize calcium burden, depth, and distribution and can guide escalation when residual mechanical resistance persists.	IVUS can improve vessel sizing and characterize plaque and calcium in selected femoropopliteal interventions.	Mechanistic/imaging parameter; DCB-specific thresholds remain unvalidated.
ISR mechanical mechanism	Intravascular imaging can identify underexpansion, undersizing, malapposition, and calcific neoatherosclerosis that should be addressed before DCB.	Applies selectively to peripheral ISR according to lesion mechanics and imaging availability.	Mechanism-directed assessment rather than a single numeric threshold.

## Data Availability

Please add the corresponding content of this part. No new data were created or analyzed in this study. Data sharing is not applicable to this article.

## References

[1] Aziz A., Bhatia G., Pitt M., Choudhury A., Hailan A., Upadhyaya S., Lee L., Testa L., Casenghi M., Ielasi A. (2021). Intravascular lithotripsy in calcified-coronary lesions: A real-world observational, European multicenter study. Catheter Cardiovasc. Interv..

[2] Basavarajaiah S., Ielasi A., Raja W., Naneishvili T., Testa L., Rubbio A.P., Mastrangelo A., Bartorelli A.L., Bhatia G., Choudhury A. (2023). Long-term outcomes following intravascular lithotripsy (IVL) for calcified coronary lesions: A Real-World Multicenter European Study. Catheter. Cardiovasc. Interv..

[3] Ielasi A., Moscarella E., Testa L., Gioffrè G., Morabito G., Cortese B., Colangelo S., Tomai F., Arioli F., Maioli M. (2020). IntravaScular Lithotripsy for the Management of UndILatable Coronary StEnt: The SMILE Registry. Cardiovasc. Revasc. Med..

[4] Tepe G., Brodmann M., Werner M., Bachinsky W., Holden A., Zeller T., Mangalmurti S., Nolte-Ernsting C., Bertolet B., Scheinert D. (2021). Intravascular Lithotripsy for Peripheral Artery Calcification: 30-Day Outcomes From the Randomized Disrupt PAD III Trial. JACC Cardiovasc. Interv..

[5] Kereiakes D.J., Di Mario C., Riley R.F., Fajadet J., Shlofmitz R.A., Saito S., Ali Z.A., Klein A.J., Price M.J., Hill J.M. (2021). Intravascular Lithotripsy for Treatment of Calcified Coronary Lesions: Patient-Level Pooled Analysis of the Disrupt CAD Studies. JACC Cardiovasc. Interv..

[6] Piraino D., Cimino G., Buccheri D., Dendramis G., Andolina G., Cortese B. (2017). Recurrent in-stent restenosis, certainty of its origin, uncertainty about treatment. Int. J. Cardiol..

[7] Alfonso F., Jeger R. (2026). Drug-Coated Balloons for In-Stent Restenosis and De Novo Coronary Lesions: State of the Art and Lessons Learned From Recent Trials. J. Soc. Cardiovasc. Angiogr. Interv..

[8] Csore J., Crichton A.B., Gu J., Benfor B., Corr S., Boateng K., Roy T.L. (2025). Developing a Scanning Electron Microscopy Approach to Evaluate Paclitaxel Deposition From Drug Coated Balloons in a Human Cadaveric Peripheral Artery Disease Model. EJVES Vasc. Forum.

[9] Konishi H., Habara M., Nasu K., Koshida R., Kinoshita Y., Tsuchikane E., Terashima M., Matsubara T., Suzuki T. (2022). Impact of Optimal Preparation Before Drug-Coated Balloon Dilatation for De Novo Lesion in Patients With Coronary Artery Disease. Cardiovasc. Revasc. Med..

[10] Lee T., Ashikaga T., Nozato T., Nagata Y., Kaneko M., Miyazaki R., Misawa T., Taomoto Y., Okata S., Nagase M. (2024). Predictors of target lesion failure after percutaneous coronary intervention with a drug-coated balloon for de novo lesions. EuroIntervention.

[11] Khialani B., Gurgoglione F.L., Hasan J., Wańha W., Oemrawsingh R.M., Cortese B. (2026). Lesion preparation before drug-coated balloon angioplasty for in-stent restenosis: Comparison between specialty and conventional balloons. REC Interv. Cardiol..

[12] Iossa L.A., Ferrone M., Salemme L., Laganà E., Pucciarelli A., Franzese M., Ciliberti G., Verdoliva S., Sgherzi G., Popusoi G. (2026). Development and Internal Validation of a Bailout Risk Score in PCI with Drug-Coated Balloons. J. Clin. Med..

[13] Tepe G., Brodmann M., Bachinsky W., Holden A., Zeller T., Mangalmurti S., Nolte-Ernsting C., Virmani R., Parikh S.A., Gray W.A. (2022). Intravascular Lithotripsy for Peripheral Artery Calcification: Mid-term Outcomes From the Randomized Disrupt PAD III Trial. J. Soc. Cardiovasc. Angiogr. Interv..

[14] Cuculi F., Bossard M., Zasada W., Moccetti F., Voskuil M., Wolfrum M., Malinowski K.P., Toggweiler S., Kobza R. (2020). Performing percutaneous coronary interventions with predilatation using non-compliant balloons at high-pressure versus conventional semi-compliant balloons: Insights from two randomised studies using optical coherence tomography. Open Heart.

[15] Lazar F.L., Onea H.L., Olinic D.M., Cortese B. (2024). A 2024 scientific update on the clinical performance of drug-coated balloons. AsiaIntervention.

[16] Gurgoglione F.L., Gattuso D., Greco A., Donelli D., Niccoli G., Cortese B. (2025). Angiographic and clinical impact of balloon inflation time in percutaneous coronary interventions with sirolimus-coated balloon: A subanalysis of the EASTBOURNE study. Cardiovasc. Revasc. Med..

[17] Fondazione Ricerca e Innovazione Cardiovascolare ETS (2026). Impact of Lesion PREParation With Modifying Balloons During Drug-coated Balloon (DCB) Angioplasty for In-stent Restenosis (ISR)—PICCOLETO XI A-PREP Study. https://clinicaltrials.gov/study/NCT07391033.

[18] Panda S., Sarkar G., Bhadra A., Mandal S. (2025). Rotational Atherectomy versus Cutting Balloon Angioplasty in Severely Calcified Coronary Lesions: A Prospec-tive Single-Center Comparative Study. Eur. J. Cardiovasc. Med..

[19] Abdel-Wahab M., Richardt G., Büttner H.J., Toelg R., Geist V., Meinertz T., Schofer J., King L., Neumann F.-J., Khattab A.A. (2013). High-speed rotational atherectomy before paclitaxel-eluting stent implantation in complex calcified coronary lesions: The randomized ROTAXUS (Rotational Atherectomy Prior to Taxus Stent Treatment for Complex Native Coronary Artery Disease) trial. JACC Cardiovasc. Interv..

[20] Abdel-Wahab M., Toelg R., Byrne R.A., Geist V., El-Mawardy M., Allali A., Rheude T., Robinson D.R., Abdelghani M., Sulimov D.S. (2018). High-Speed Rotational Atherectomy Versus Modified Balloons Prior to Drug-Eluting Stent Implantation in Severely Calcified Coronary Lesions. Circ. Cardiovasc. Interv..

[21] Lee M.S., Martinsen B.J., Shlofmitz R., Shlofmitz E., Lee A.C., Chambers J. (2017). Orbital atherectomy treatment of severely calcified coronary lesions in patients with impaired left ventricular ejection fraction: One-year outcomes from the ORBIT II study. EuroIntervention.

[22] Kirtane A.J., Généreux P., Lewis B., Shlofmitz R.A., Dohad S., Choudary J., Dahle T., Pineda A.M., Shunk K., Maehara A. (2025). Orbital atherectomy versus balloon angioplasty before drug-eluting stent implantation in severely calcified lesions eligible for both treatment strategies (ECLIPSE): A multicentre, open-label, randomised trial. Lancet.

[23] Benedetto D., Frazzetto M., Regazzoli D., Bezubka J., Fischetti D., Jones D., Khialani B., Minha S., Ugo F., Wańczura P. (2025). TCT-164 Coronary AtheRectomy and Drug Coated Ballon Intervention in All-comer Calcific lesions: The CARDIAC Trial. JACC.

[24] Kereiakes D.J., Hill J.M., Shlofmitz R.A., Klein A.J., Riley R.F., Price M.J., Herrmann H.C., Bachinsky W., Waksman R., Stone G.W. (2022). Intravascular Lithotripsy for Treatment of Severely Calcified Coronary Lesions: 1-Year Results From the Disrupt CAD III Study. J. Soc. Cardiovasc. Angiogr. Interv..

[25] Wańha W., Wolny R., Kleczyński P., Mattesini A., Iwańczyk S., Dziarmaga M., Kulczycki J., Kuźma Ł., Gąsior P., Wańczura P. (2025). Acute and short-term outcome of Intravascular Coronary Lithotripsy and Excimer Laser Coronary Atherectomy for Severe Stent Underexpansion: The Multicenter IVL-ELCA DRAGON Registry. Kardiol. Pol..

[26] Dash D., Mody R., Dash U., Hasanfatta N. (2025). Managing Balloon-Uncrossable and Undilatable Coronary Lesions: Unravelling the Mystique. J. Cardiovasc. Interv..

[27] Antoniades S., Korosoglou G. (2026). The Role of Atherectomy in Lesion Preparation for Peripheral Artery Disease: Evolution of Technologies, Available Devices, and Contemporary Evidence. Rev. Cardiovasc. Med..

[28] Dippel E.J., Makam P., Kovach R., George J.C., Patlola R., Metzger D.C., Mena-Hurtado C., Beasley R., Soukas P., Colon-Hernandez P.J. (2015). Randomized controlled study of excimer laser atherectomy for treatment of femoropopliteal in-stent restenosis: Initial results from the EXCITE ISR trial (EXCImer Laser Randomized Controlled Study for Treatment of FemoropopliTEal In-Stent Restenosis). JACC Cardiovasc. Interv..

[29] Shibui T., Tsuchiyama T., Masuda S., Nagamine S. (2021). Excimer laser coronary atherectomy prior to paclitaxel-coated balloon angioplasty for de novo coronary artery lesions. Lasers Med. Sci..

[30] Zeller T., Langhoff R., Rocha-Singh K.J., Jaff M.R., Blessing E., Amann-Vesti B., Krzanowski M., Peeters P., Scheinert D., Torsello G. (2017). Directional Atherectomy Followed by a Paclitaxel-Coated Balloon to Inhibit Restenosis and Maintain Vessel Patency: Twelve-Month Results of the DEFINITIVE AR Study. Circ. Cardiovasc. Interv..

[31] Yiu J., Tippireddy R., Biasi L., Patel S., Saha P., Saratzis A., Katsanos K., Zayed H. (2025). Systematic Review and Network Meta-analysis of Vessel Preparation Techniques With Plain Balloon Angioplasty, Atherectomy, or Intravascular Lithotripsy Before Application of a Drug Coated Balloon to Treat Atherosclerotic Femoropopliteal Disease. Eur. J. Vasc. Endovasc. Surg..

[32] Basavarajaiah S., Sharma V., Agip-Fustamante D., Freire S.J.C., El-Mokdad R., Bhatia G., Waduge B.H.L., Ielasi A., Fernández J.F.D., Athukorala S. (2023). Which predilatation balloons provide the best lesion preparation prior to use of drug coated balloons in de-novo lesions? Results from the PREPARE study. Minerva Cardiol. Angiol..

[33] Colletti G., Ruzsa Z., Gach O., Jossart A., Peter L., Spanò A., Cocco N., Boukhris M., Ungureanu C. (2026). Calcium modifying dedicated balloons: A contemporary review. Front. Cardiovasc. Med..

[34] Tokuda T., Yoshioka N., Tanaka A., Kojima S., Yamaguchi K., Yanagiuchi T., Ogata K., Takei T., Nakama T. (2026). Clinical Impacts and Procedural Risks of Rotational Atherectomy for Heavily Calcified Femoropopliteal Lesions. JACC Asia.

[35] Kufner S., Joner M., Schneider S., Tölg R., Zrenner B., Repp J., Starkmann A., Xhepa E., Ibrahim T., Cassese S. (2017). Neointimal Modification With Scoring Balloon and Efficacy of Drug-Coated Balloon Therapy in Patients With Restenosis in Drug-Eluting Coronary Stents: A Randomized Controlled Trial. JACC Cardiovasc. Interv..

[36] Tepe G., Laird J., Schneider P., Brodmann M., Krishnan P., Micari A., Metzger C., Scheinert D., Zeller T., Cohen D.J. (2015). Drug-Coated Balloon Versus Standard Percutaneous Transluminal Angioplasty for the Treatment of Superficial Femoral and Popliteal Peripheral Artery Disease. Circulation.

[37] Rosenfield K., Jaff M.R., White C.J., Rocha-Singh K., Mena-Hurtado C., Metzger D.C., Brodmann M., Pilger E., Zeller T., Krishnan P. (2015). Trial of a Paclitaxel-Coated Balloon for Femoropopliteal Artery Disease. N Engl. J. Med..

[38] Tomoi Y., Soga Y., Hirano T., Tsunoda K., Ando K. (2025). Long-Term Clinical Impact of Successful Vessel Preparation in Femoropopliteal Drug-Coated Balloon Angioplasty. Catheter Cardiovasc. Interv..

[39] Gitto M., Gabrielli A., Leone P.P., Sanz-Sanchez J., Tartaglia F., Bernardini V., Regazzoli D., Mangieri A., Reimers B., Latib A. (2026). Drug-coated balloons in complex large-vessel coronary artery disease: A comprehensive review of current evidence and future perspectives. Front. Cardiovasc. Med..

[40] Oliveri F., van Oort M.J., Phagu A.A., Al Amri I., Bingen B.O., Paradies V., Mincione G., Claessen B.E., Dimitriu-Leen A.C., Kefer J. (2025). Drug-Coated Balloon After Intravascular Lithotripsy: Procedural and Clinical Outcome of the “Crack and Drug” Strategy. JACC Adv..

[41] Tupayachi D.O. (2026). EuroPCR 2026|Does Calcium Morphology Define the Choice Between IVL and Cutting Balloon?. https://solaci.org/en/2026/05/20/europcr-2026-does-calcium-morphology-define-the-choice-between-ivl-and-cutting-balloon/.

[42] Oliveri F., Van Oort M.J., Al Amri I., Bingen B.O., Claessen B.E., Dimitriu-Leen A.C., Kefer J., Girgis H., Vossenberg T., Van der Kley F. (2025). Coronary Compliance Modification by Intravascular Lithotripsy: New Predictor of Stent Expansion in Calcified Coronary Lesions. J. Soc. Cardiovasc. Angiogr. Interv..

[43] Kristensen A.T., Christiansen E.H., Holm N.R., Kelbæk H., Engstrøm T., Veien K., Raungaard B., Kleveland O., Bennett J., Laanmets P. (2026). Balloon Lithotripsy Added to Conventional Preparation Before Stent Implantation in Severely Calcified Coronary Lesions. JACC Cardiovasc. Interv..

[44] Gao X.-F., Ge Z., Kong X.-Q., Chen X., Han L., Qian X.-S., Zuo G.-F., Wang Z.-M., Wang J., Song J.-X. (2024). Intravascular Ultrasound vs Angiography-Guided Drug-Coated Balloon Angioplasty: The ULTIMATE III Trial. JACC Cardiovasc. Interv..

[45] Ninomiya K., Serruys P.W., Colombo A., Reimers B., Basavarajaiah S., Sharif F., Testa L., Di Mario C., Nerla R., Ding D. (2023). A Prospective Randomized Trial Comparing Sirolimus-Coated Balloon With Paclitaxel-Coated Balloon in De Novo Small Vessels. JACC Cardiovasc. Interv..

[46] Ninomiya K., Serruys P., Masuda S., Huang J., Daixin D., Kotoku N., Kageyama S., Doshi M., Wijns W., Tu S. (2023). CRT-600.05 Precise Assessments of Dissection Following Drug-Coated Balloon Treatment and Its Impact on Clinical Outcomes in De Novo Small Vessel Disease. JACC Cardiovasc. Interv..

[47] Tobe A., Serruys P., Miyashita K., Oshima A., Revaiah P.C., Tsai T.-Y., Jouke D., Garg S., McInerney A., Onuma Y. (2025). Serial quantitative optical coherence tomography for luminal volume changes following either paclitaxel or sirolimus coated balloon in de novo small coronary artery lesions. Cardiovasc. Revasc. Med..

[48] Luan S., Yu X., Lei S., Ma C., Wang X., Xue X., Ding Y., Ma T., Zhu B. (2023). Deep learning for fast super-resolution ultrasound microvessel imaging. Phys. Med. Biol..

[49] Song P., Rubin J.M., Lowerison M.R. (2023). Super-resolution ultrasound microvascular imaging: Is it ready for clinical use?. Z. Med. Phys..

[50] Cortese B., Granada J.F., Scheller B., Schneider P.A., Tepe G., Scheinert D., Garcia L., Stabile E., Alfonso F., Ansel G. (2016). Drug-coated balloon treatment for lower extremity vascular disease intervention: An international positioning document. Eur. Heart J..

[51] Korosoglou G., Schmidt A., Lichtenberg M., Malyar N., Stavroulakis K., Reinecke H., Grözinger G., Patrone L., Varcoe R.L., Soukas P.A. (2025). Global Algorithm for the Endovascular Treatment of Chronic Femoropopliteal Lesions: An Interdisciplinary Expert Opinion Statement. JACC Cardiovasc. Interv..

[52] Allan R.B., Puckridge P.J., Spark J.I., Delaney C.L. (2022). The Impact of Intravascular Ultrasound on Femoropopliteal Artery Endovascular Interventions: A Randomized Controlled Trial. JACC Cardiovasc. Interv..

[53] Wardle B.G., Ambler G.K., Radwan R.W., Hinchliffe R.J., Twine C.P. (2020). Atherectomy for peripheral arterial disease. Cochrane Database Syst. Rev..

